# Development of gross motor skills in children under the age of 3 years: a decision tree approach

**DOI:** 10.3389/fpubh.2024.1421173

**Published:** 2024-10-22

**Authors:** Yuxiang Xiong, Xuhuai Hu, Jindan Cao, Li Shang, Yibei Yao, Ben Niu

**Affiliations:** ^1^Department of Medical Informatics, School of Public Health, Jilin University, Changchun, China; ^2^Shenzhen Health Development Research and Data Management Center, Shenzhen, China; ^3^Department of Software Technology, School of Computer Science and Software Engineering, Shenzhen Institute of Information Technology, Shenzhen, China; ^4^Department of Management Science, College of Management, Shenzhen University, Shenzhen, China

**Keywords:** gross motor, decision tree, under the age of 3 years, stratified random sampling, feature importance

## Abstract

**Background:**

The early years of life are critical for gross motor development (GMD). This study utilized decision tree modeling to examine the influences on gross motor development in children under the age of 3 years and to rank the key factors affecting their development.

**Methods:**

Based on randomized stratified sampling, 9,507 children aged 0–3 years in Shenzhen were included in this study. The Ages and Stages Questionnaires (ASQ) were utilized for the assessment of gross motor development. The chi-square test was used to compare groups, and variables were screened using univariable and multivariable regression analyses. Decision tree modeling was employed to rank the importance of statistically significant variables.

**Results:**

The research found a prevalence of gross motor developmental delay of 1.41% among the respondents. The accuracy of the decision tree model is 70.96%. The results demonstrated a strong correlation between seven variables affecting the gross motor development of children, which were ranked based on importance: age, whether to provide supplementary food, average time spent interacting with children, family type, feeding method, mode of delivery, and birth order.

**Conclusion:**

The risk of gross motor developmental delay increases with age. Furthermore, supplementary food and interacting with other children are critical factors in improving children’s GMD delay. It is therefore imperative to enhance the monitoring of children’s gross motor skills through regular developmental assessments that detect potential GMD delays. Moreover, family type, feeding method, mode of delivery, and birth order were also predictive factors of GMD delay.

## 1 Introduction

Building gross motor skills (GMS) is an essential component of the motor growth of the body. Gross movements result from the actions of large muscle groups, whereas fine movements happen through the actions of smaller muscles or muscle groups. The combination of gross and fine movements constitutes human behavior of practical significance. Important gross movements in childhood include various infant reflexes (e.g., stepping and crawling reflexes), autonomous mobility (e.g., creeping and rolling), basic motor skills (e.g., running and jumping), and object manipulation abilities (e.g., throwing and kicking) (1). The initial 1,000 days of life are crucial for children’s growth and development, significantly influencing their future (2). Moreover, GMS will facilitate experience, discovery, learning, and development during this life stage. In this regard, GMS is positively correlated with children’s physical development, cognitive development (3, 4), cardiometabolic risk (5), and social skills (6).

Among the typical gross motor evaluation instruments [e.g., Alberta Infant Motor Scale (AIMS) (7), Bruininks–Oseretsky Test of Motor Proficiency-2 (BOT-2), Bayley Scale of Infant and Toddler Development-III (Bayley-III), Movement Assessment Battery for Children-2 (MABC-2), McCarron Assessment of Neuromuscular Development (MAND), Neurological Sensory Motor Developmental Assessment (NSMDA), Peabody Developmental Motor Scales-2 (PDMS-2), and Test of Gross Motor Development-2 (TGMD-2)] that are widely employed in the detection, diagnosis, and assessment of pediatric movement disorders (8), the Bayley-III is the most commonly utilized instrument for diagnosing developmental delay in children under the age of 3 years. Nevertheless, several challenges exist in the implementation of this tool in regions and countries with limited resources. As an effective solution, the Ages and Stages Questionnaire (ASQ) serves as a simple and cost-effective screening tool that allows parents or caregivers to complete (9). The ASQ has been extensively used in various countries and has emerged as a commonly adopted screening instrument for child development globally (10–12). Using the ASQ, a Peruvian study assessed five major areas of child development: communication skills, gross motor abilities, fine motor skills, problem-solving capabilities, and personal–social competencies (13). A study from Argentina employed the Ages and Stages Questionnaires—Third Edition (ASQ-3) and found that 19.5% of the surveyed children were at risk for neurodevelopmental conditions. The ASQ-3 demonstrated congruence in psychometric characteristics when compared to the National Screening Test (14).

To devise effective strategies for preventing gross motor developmental delay, it is essential to comprehend the underlying risk factors. Research involving 205 toddlers indicates that encouraging extended and regular nighttime sleep, in conjunction with an earlier bedtime, could enhance the development of gross motor skills (15). Further research implies a positive correlation between gross motor skill proficiency and physical fitness in Chilean children aged 4–6 years (16). A cross-sectional descriptive analysis reveals that preschool children in South Africa, regardless of their economic backgrounds, exhibit high levels of gross motor skill proficiency (17). Furthermore, existing literature indicates that GMS is positively associated with physical activity and fitness (18), perceived competence (19), and body weight (20). Another cross-sectional investigation found no correlation between physical activity levels and Australian toddlers, highlighting the importance of a specific age for promoting physical activity (21).

Although several studies have probed the factors associated with developmental delay, each presents its own limitations. The majority of studies primarily examined cohorts before the year 2000, and the geographic scope of data collection was predominantly constrained, which may diminish the generalizability of their findings (22–26). Moreover, few studies have thoroughly investigated the current state of gross motor development in Shenzhen for children aged 0–3 years.

Bronfenbrenner’s ecological theory offers a framework for analyzing children’s gross motor developmental delays and their association with potential risk factors. It assumes that children’s development is the result of environmental influences and that their interaction with the environment is marked by complexity and multidimensionality (27). The environment in which children develop is divided into four levels: micro, mediated, outer, and macro. Each level contains positive or negative factors that influence children’s growing performance (28). Children are affected by direct interactions and the broader environment, including family, community, and society (29). The Dynamic Systems Theory provides a framework that defines behavior as an emergent outcome of a self-regulating, multifaceted system that evolves over time, underscoring the interplay among various elements as the impetus for behavioral and developmental changes (30). This study explored the influencing factors of GMS at the individual and family levels. A decision tree was used to analyze the importance of its influencing factors. The findings of our study offer an empirical reference for the prevention and control of gross motor developmental delay.

## 2 Methods

### 2.1 Study population and sample

The study involved 9,507 infants and toddlers aged 0–3 years who received health examinations at the Shenzhen Social Health Center’s Child Health Clinic between August 2021 and June 2022. The survey was conducted by pediatric healthcare professionals who had undergone specialized training and met the qualification criteria. The project personnel monitored the evaluation data throughout the entire process, while the questionnaire and data compiled by the data quality control personnel were reviewed on the same day. All participating parents provided basic information and completed the ASQ-3 questionnaire.

Exclusion criteria included the following: (1) children exhibiting developmental delays or severe hereditary conditions; (2) caregivers with cognitive deficits or emotional issues; (3) children over 36 months or those who refused informed consent; and (4) the involvement of non-primary caregivers during the evaluation.

### 2.2 Measures

We utilized electronic resources such as CNKI and PubMed to review the domestic and international literature on the developmental levels of children aged 0–3 years and their influencing factors and screened possible influencing factors for inclusion in the study based on previous reports in the literature.

#### 2.2.1 Dependent variable

GMS of infants and toddlers aged 0–3 years were screened and assessed using the ASQ-3. The Ages and Stages Questionnaire—Third Edition—Gross Motor domain (ASQ-3-GM) score is valid to identify gross motor developmental delay in young children (12). Scholars introduced the ASQ-3 to China, created the ASQ-Chinese (ASQ-C), and collaborated with the authors of the ASQ system to examine the national norms and psychometric properties of Chinese children aged 1–66 months. The ASQ-C demonstrated a sensitivity of 87.50% and a specificity of 84.48% (31).

The ASQ-3 is a parent-completed developmental screening tool comprising 21 age-specific questionnaires for children aged 0–66 months. All questionnaires include six questions in each of five developmental domains: gross motor, fine motor, problem-solving, personal-social, and communication. Responses to each question are categorized as ‘yes’ (10 points), ‘sometimes’ (5 points), or ‘not yet’ (0 points) f, yielding a maximum score of 60 in each domain. Domain scores are compared to normative scores, with cut-off points categorizing development in that domain as ‘typical’ (<1SD below the mean), ‘monitor’ (≥1SD to <2SD below the mean), or ‘refer for further assessment’ (‘refer’) (≥2SD below the mean). According to ASQ-3 instructions, children with ASQ-3-GM scores below the ‘refer’ cutoff require further assessment for gross motor delay (12, 32).

#### 2.2.2 Independent variables

##### 2.2.2.1 Basic information

The items were composed of age (months), sex, gestational week, birth weight, and birth order. Age was divided into three categories (1 = 1–12 months, 2 = 13–24 months, and 3 = 25–36 months). Sex was categorized as male and female (0 = male and 1 = female). Using 37 weeks as the cut-off point, the gestation week was categorized into preterm and non-preterm infants (1 = <37, 2 = ≥37). Birth weight was categorized as low birth weight, normal weight, and macrosomia (1 = low birth weight, 2 = normal weight and 3 = macrosomia). The order of birth was categorized into two types depending on whether the child was the first birth (1 = first-born child and 2 = non-first-born child).

##### 2.2.2.2 Feeding situation

By asking parents how their babies were fed within the age of 6 months, they were categorized as exclusively breastfeeding (fed only mother’s milk without any other dairy products or animal milk added), mixed feeding (breastfed with formula milk), and bottle-feeding (fed with formula milk only). The mode of delivery is categorized as natural delivery, cesarean section, and instrumental delivery (including forceps or suction). Feeding status was determined by asking parents “whether or not complementary foods have been added” and “Is the baby getting colostrum?”(0 = No; 1 = Yes).

##### 2.2.2.3 Living habits

Average parent–child reading time per day (1 = less than 5 min, 2 = 5–15 min, 3 = 16–30 min, and 4 = more than 30 min). Average time spent interacting with other children per day (1 = less than 15 min, 2 = 15–30 min, 3 = 31–60 min, and 4 = more than 60 min). Average time spent outdoors per day (1 = less than 30 min, 2 = 30–60 min, 3 = 61–90 min, 4 = 91–120 min, and 5 = more than 60 min).

##### 2.2.2.4 Parental information

Parental education level (1 = middle school and below, 2 = high school, and 3 = college), mother’s age at childbearing (1 = <35, 2 = ≥35), father’s age at childbearing (1 = ≤24, 24 < 2 ≤ 34, 3 > 34), maternal employment status (0 = unemployment, 1 = employment), career type of the parents (1 = government/institutional cadres/civil servants, 2 = professionals and technicians, 3 = clerks and service workers, 4 = production and manufacturing workers, 5 = others, and 6 = unemployment), and maternal health status during pregnancy (gestational diabetes mellitus, hypertension during pregnancy, anemia during pregnancy, bacterial vaginitis (BVI), placenta previa and prenatal depression) were collected. Family type was categorized as follows: nuclear family (consisting of married couples and unmarried or adopted children), a backbone family (consisting of three generations of parents, married children, and their offspring), and other family structures.

### 2.3 Decision tree

The decision tree model of machine learning was created using the open-source software R Studio. The decision tree has the following advantages: (1) Decision-tree algorithms are adept at generating straightforward and interpretable classification guidelines, displaying these rules in a clear, top-down graphical format. (2) These models are proficient at managing intricate interactions among predictor variables as evidenced by their capacity to accommodate high multicollinearity (33). In this study, a decision tree model was developed and the significant variables were ranked in order of importance.

### 2.4 Statistical analysis

Categorical variables are expressed as frequencies and percentages, and the difference between groups was tested using the chi-square test. The data set was randomly divided into two parts: 70% as the training set and 30% as the validation set. The variables were screened using univariable and multivariable regression analyses (*p* < 0.1); all tests were two-tailed. Odds ratios (ORs) and 95% confidence intervals (CIs) were used to quantify the associations between factors and gross motor developmental delay. Given the significant imbalance in this data set, the ROSE algorithm was used to address the issue, and a decision tree was constructed based on the processed data. The statistical analysis and decision tree analysis were performed using the R software package (version 4.3.1).

## 3 Results

### 3.1 Descriptive analysis

Table 1 presents the baseline characteristics of all participants grouped according to the presence or absence of developmental delay. Approximately 1.43% of children under 3 years of age have delayed gross motor development. One-half of respondents were under 12 months, of which 43.77% were female. The proportion of first-born infants reached 55.45%. Significant differences (*p*<0.05) were noted in variables such as age, mode of delivery, complementary food, mother’s education, father’s education, family type and average time spent interacting with others children when comparing the typical development group to the atypical development (Table 1).

**Table 1:** Sample characteristics and prevalence of gross motor developmental delay.

Variable	Total (*N* = 9,507)	Typical development (*N* = 9,373)	Atypical development (*N* = 134)	*p*-value
Age (months)				0.001
1–12	5,575 (58.64%)	5,511 (58.80%)	64 (47.76%)	
13–24	2,650 (27.87%)	2,614 (27.89%)	36 (26.87%)	
25–36	1,282 (13.48%)	1,248 (13.31%)	34 (25.37%)	
Sex				0.772
Male	5,346 (56.23%)	5,269 (56.21%)	77 (57.46%)	
Female	4,161 (43.77%)	4,104 (43.79%)	57 (42.54%)	
Gestation period				0.904
<37	519 (5.46%)	512 (5.46%)	7 (5.22%)	
≥37	8,988 (94.54%)	8,861 (94.54%)	127 (94.78%)	
Birth order				0.061
First-born child	5,272 (55.45%)	5,187 (55.34%)	85 (63.43%)	
Non-first-born child	4,235 (44.55%)	4,186 (44.66%)	49 (36.57%)	
Feeding method				0.080
Exclusive breastfeeding	4,676 (49.18%)	4,623 (49.32%)	53 (39.55%)	
Mixed feeding	3,751 (39.46%)	3,688 (39.35%)	63 (47.01%)	
Artificial feeding	1,080 (11.36%)	1,062 (11.33%)	18 (13.43%)	
Mode of delivery				0.011
Natural delivery	6,247 (65.71%)	6,160 (65.72%)	87 (64.93%)	
Cesarean section	3,087 (32.47%)	3,047 (32.51%)	40 (29.85%)	
Instrumental delivery	173 (1.82%)	166 (1.77%)	7 (5.22%)	
Whether supplementary food was given				<0.001
No	2,007 (21.11%)	1,960 (20.91%)	47 (35.07%)	
Yes	7,500 (78.89%)	7,413 (79.09%)	87 (64.93%)	
Whether colostrum was consumed				0.608
No	1,352 (14.22%)	1,335 (14.24%)	17 (12.69%)	
Yes	8,155 (85.78%)	8,038 (85.76%)	117 (87.31%)	0.608
Whether the mother suffered from prenatal depression during pregnancy and childbirth				0.084
No	9,492 (99.84%)	9,359 (99.85%)	133 (99.25%)	
Yes	15 (0.16%)	14 (0.15%)	1 (0.75%)	
Whether the mother had BVI during pregnancy and childbirth				
No	9,390 (98.77%)	9,257 (98.76%)	133 (99.25%)	
Yes	117 (1.23%)	116 (1.24%)	1 (0.75%)	
Mother’s education				<0.001
Junior high school	2,282 (24.00%)	2,236 (23.86%)	46 (34.33%)	
High school	1,838 (19.33%)	1,805 (19.26%)	33 (24.63%)	
College	5,387 (56.66%)	5,332 (56.89%)	55 (41.04%)	
Father’s education				0.007
Junior high school	1,955 (20.56%)	1,928 (20.57%)	27 (20.15%)	
High school	2,088 (21.96%)	2,044 (21.81%)	44 (32.84%)	
College	5,464 (57.47%)	5,401 (57.62%)	63 (47.01%)	
Whether the mother is in active status				0.131
Unemployment	4,353 (45.79%)	4,283 (45.70%)	70 (52.24%)	
Employment	5,154 (54.21%)	5,090 (54.30%)	64 (47.76%)	
Mother’s age at childbearing				0.918
<35	8,201 (86.26%)	8,085 (86.26%)	116 (86.57%)	
≥35	1,306 (13.74%)	1,288 (13.74%)	18 (13.43%)	
Father’s age at childbearing				0.435
≤24	416 (4.38%)	413 (4.41%)	3 (2.24%)	
(24, 34]	6,192 (65.13%)	6,105 (65.13%)	87 (64.93%)	
>34	2,899 (30.49%)	2,855 (30.46%)	44 (32.84%)	
Family type				0.005
Nuclear family	4,509 (47.43%)	4,428 (47.24%)	81 (60.45%)	
Backbone family	4,437 (46.67%)	4,393 (46.87%)	44 (32.84%)	
Other family structures	561 (5.90%)	552 (5.89%)	9 (6.72%)	
Average parent–child reading time per day				0.356
Less than 5 min	3,183 (33.48%)	3,129 (33.38%)	54 (40.30%)	
5–15 min	3,262 (34.31%)	3,218 (34.33%)	44 (32.84%)	
16–30 min	1,940 (20.41%)	1,918 (20.46%)	22 (16.42%)	
Average time spent daily interacting with children	1,122 (11.80%)	1,108 (11.82%)	14 (10.45%)	
Less than 30 min				<0.001
15–30 min	823 (8.66%)	802 (8.56%)	21 (15.67%)	
31–60 min	1,949 (20.50%)	1,910 (20.38%)	39 (29.10%)	
More than 60 min	2,639 (27.76%)	2,602 (27.76%)	37 (27.61%)	
Average time spent outdoors per day	4,096 (43.08%)	4,059 (43.31%)	37 (27.61%)	
Less than 30 min				0.265
30–60 min	1,128 (11.86%)	1,105 (11.79%)	23 (17.16%)	
61–90 min	2,476 (26.04%)	2,442 (26.05%)	34 (25.37%)	
91–120 min	2,334 (24.55%)	2,305 (24.59%)	29 (21.64%)	
More than 120 min	1,697 (17.85%)	1,670 (17.82%)	27 (20.15%)	

### 3.2 Results of logistic regression

To investigate more deeply the relationship between various factors and gross motor retardation, we used logistics regression to screen for statistically significant variables. The results of the univariate regression analysis indicated that age, birth order, feeding method, mode of delivery, whether supplementary food was given, family type, and average time spent interacting with children were related to GMS (*p* < 0.1). The results of multivariate regression analysis indicated that with age, the likelihood of gross motor developmental delay increased relative to the probability of not occurring (OR = 2.8, 95% CI = [1.486, 5.274] and OR = 6.635, 95% CI = [1.486, 5.274]). Within the age of 6 months, mixed feeding had an increased risk of gross motor developmental delay compared to exclusive breastfeeding (OR = 1.684, 95% CI = [1.079, 2.629]). There was an increased prevalence of gross motor developmental delay in children with instrumental delivery compared to natural delivery (OR = 3.65, 95% CI = [1.489, 8.95]) (Table 2).

**Table 2:** Results of the logistic regression analysis on the training set.

Variable	Typical development	Atypical development	Univariate logistics regression		Multivariate logistics regression	
			OR (95%CI)	*p*	OR (95%CI)	*p*
Age (months)						
1–12	3,871 (59%)	41 (43.6%)	Ref		Ref	
13–24	1,819 (27.7%)	25 (26.6%)	1.298 (0.787, 2.14)	0.3076	2.8 (1.486, 5.274)	0.0014
25–36	872 (13.3%)	28 (29.8%)	3.032 (1.865, 4.929)	<0.001	6.635 (3.518, 12.514)	<0.001
Sex						
Male	3,678 (56%)	53 (56.4%)	Ref			
Female	2,884 (44%)	41 (43.6%)	0.987 (0.654, 1.487)	0.9485		
Gestation period						
<37	341 (5.2%)	7 (7.4%)	Ref			
≥37	6,221 (94.8%)	87 (92.6%)	0.681 (0.313, 1.483)	0.3334		
Birth order						
First-born child	3,651 (55.6%)	61 (64.9%)	Ref		Ref	
Non-first-born child	2,911 (44.4%)	33 (35.1%)	0.679 (0.443, 1.039)	0.0746	0.652 (0.42, 1.012)	0.0566
Feeding method						
Exclusive breastfeeding	3,240 (49.4%)	36 (38.3%)	Ref		Ref	
Mixed feeding	2,583 (39.4%)	47 (50%)	1.638 (1.058, 2.536)	0.027	1.684 (1.079, 2.629)	0.0218
Artificial feeding	739 (11.3%)	11 (11.7%)	1.34 (0.679, 2.644)	0.3993	1.212 (0.606, 2.425)	0.5867
Mode of delivery						
Natural delivery	4,297 (65.5%)	60 (63.8%)	Ref		Ref	
Cesarean section	2,153 (32.8%)	28 (29.8%)	0.931 (0.593, 1.463)	0.7577	1.029 (0.649, 1.633)	0.902
Instrumental delivery	112 (1.7%)	6 (6.4%)	3.837 (1.624, 9.066)	0.0022	3.65 (1.489, 8.95)	0.0047
Whether to provide supplementary food						
No	1,388 (21.2%)	32 (34%)	Ref		Ref	
Yes	5,174 (78.8%)	62 (66%)	0.52 (0.338, 0.8)	0.0029	0.238 (0.13, 0.436)	<0.001
Whether colostrum was consumed						
No	921 (14%)	13 (13.8%)	Ref			
Yes	5,641 (86%)	81 (86.2%)	1.017 (0.564, 1.835)	0.9546		
Whether the mother suffered from prenatal depression during pregnancy and childbirth						
No	6,552 (99.8%)	93 (98.9%)	Ref			
Yes	10 (0.2%)	1 (1.1%)	7.045 (0.893, 55.599)	0.064		
Mother’s education						
Junior high school	1,593 (24.3%)	31 (33%)	Ref		Ref	
High school	1,265 (19.3%)	27 (28.7%)	1.097 (0.651, 1.847)	0.7282	0.865 (0.307, 2.439)	0.7836
College	3,704 (56.4%)	36 (38.3%)	0.499 (0.308, 0.81)	0.0049	0.897 (0.373, 2.157)	0.8083
Father’s education						
Junior high school	1,358 (20.7%)	22 (23.4%)	Ref		Ref	
High school	1,447 (22.1%)	33 (35.1%)	1.408 (0.817, 2.427)	0.2183	1.455 (0.796, 2.661)	0.223
College	3,757 (57.3%)	39 (41.5%)	0.641 (0.379, 1.085)	0.0974	1.138 (0.569, 2.274)	0.7149
Whether the mother is in active status						
Unemployment	3,018 (46%)	51 (54.3%)	Ref			
Employment	3,544 (54%)	43 (45.7%)	0.718 (0.477, 1.08)	0.1121		
Mother’s age at childbearing						
<35	5,642 (86%)	82 (87.2%)	Ref			
≥35	920 (14%)	12 (12.8%)	0.897 (0.488, 1.651)	0.728		
Father’s age at childbearing						
≤24	290 (4.4%)	3 (3.2%)	Ref			
(24, 34]	4,257 (64.9%)	60 (63.8%)	1.362 (0.425, 4.371)	0.603		
>34	2,015 (30.7%)	31 (33%)	1.487 (0.452, 4.896)	0.5138		
Family type						
Nuclear family	3,145 (47.9%)	57 (60.6%)	Ref		Ref	
Backbone family	3,030 (46.2%)	30 (31.9%)	0.546 (0.35, 0.852)	0.0077	0.594 (0.367, 0.961)	0.0339
Other family structures	387 (5.9%)	7 (7.4%)	0.998 (0.452, 2.204)	0.9961	1.08 (0.482, 2.422)	0.8517
Average parent–child reading time per day						
Less than 5 min	2,166 (33%)	37 (39.4%)	Ref			
5–15 min	2,277 (34.7%)	27 (28.7%)	0.694 (0.421, 1.144)	0.1521		
16–30 min	1,347 (20.5%)	17 (18.1%)	0.739 (0.414, 1.317)	0.3049		
Average time spent daily interacting with children						
Less than 30 min	582 (8.9%)	13 (13.8%)	Ref		Ref	
15–30 min	1,319 (20.1%)	29 (30.9%)	0.984 (0.508, 1.907)	0.9626	0.993 (0.501, 1.967)	0.983
31–60 min	1,809 (27.6%)	25 (26.6%)	0.619 (0.314, 1.217)	0.1643	0.59 (0.287, 1.21)	0.1499
More than 60 min	2,852 (43.5%)	27 (28.7%)	0.424 (0.217, 0.826)	0.0117	0.496 (0.245, 1.004)	0.0513
Average time spent outdoors per day						
Less than 30 min	790 (12%)	17 (18.1%)	Ref			
30–60 min	1,723 (26.3%)	23 (24.5%)	0.62 (0.33, 1.168)	0.139		
61–90 min	1,609 (24.5%)	20 (21.3%)	0.578 (0.301, 1.109)	0.0991		
91–120 min	1,167 (17.8%)	17 (18.1%)	0.677 (0.344, 1.334)	0.2596		
More than 120 min	1,273 (19.4%)	17 (18.1%)	0.621 (0.315, 1.223)	0.1679		

### 3.3 Predictive analysis of gross motor developmental delay

**Figure 1:**
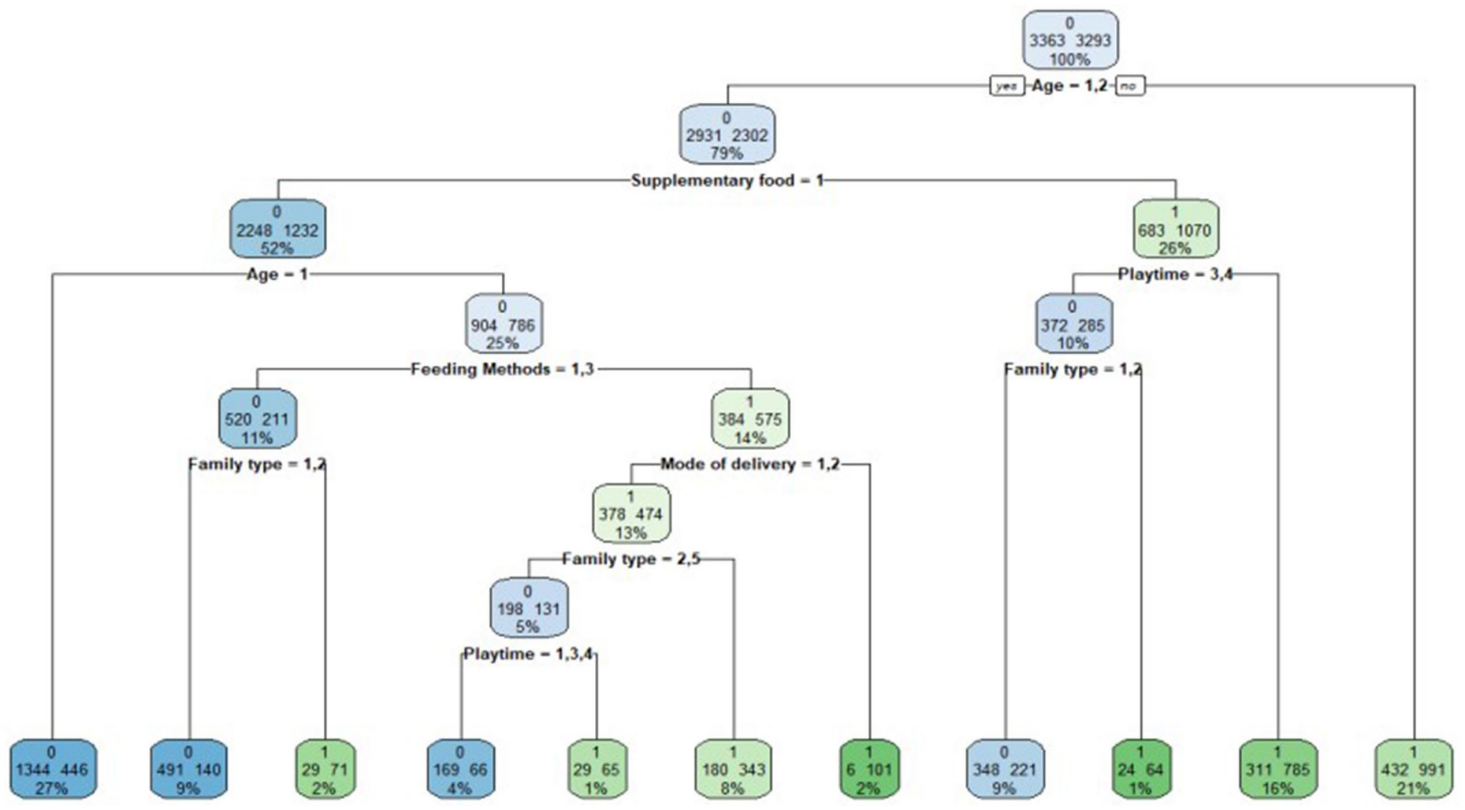
Decision tree for predicting gross motor development. The decision tree model shows that GMS are predicted to be typical development or atypical development depending on age, whether to provide supplementary food, average time spent interacting with children,family type, feeding method, mode of delivery and birth order.

**Figure 2:**
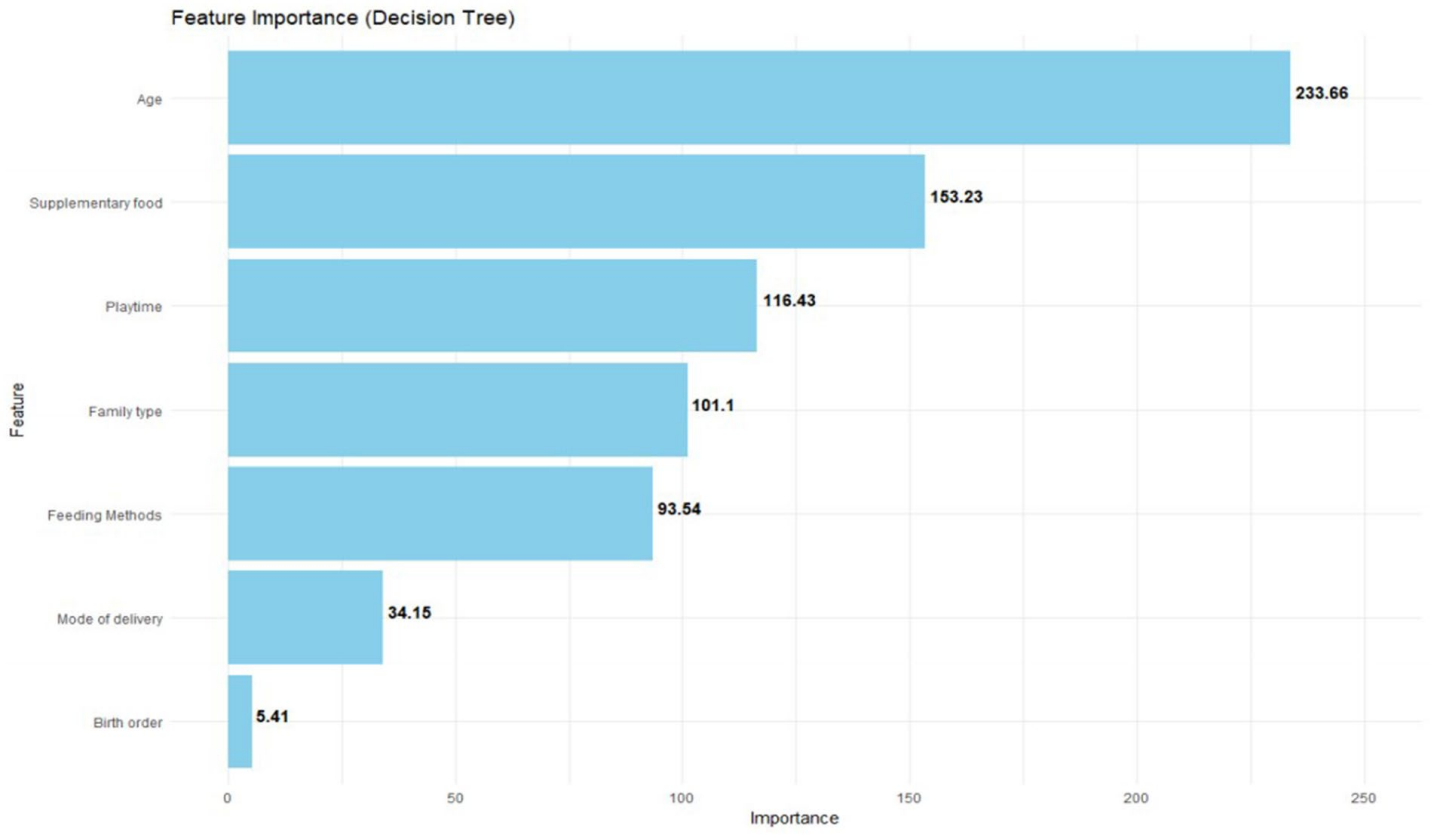
Predictor variables of gross motor developmental delay.

According to Figure 1, the predictor variable of gross motion retardation is age, supplementary food, playtime, family type, feeding method, mode of delivery, and birth order. According to Figure 2, a total of seven variables that were important for gross motor developmental delay of children under the age of 3 years were screened. These variables were prioritized based on their significance: age (importance = 233.66), supplementary food (153.23), playtime (116.43), family type (101.1), feeding method (93.54), mode of delivery (34.15), and birth order (5.41).

### 3.4 Evaluation of decision tree model

The data set was divided into a training set and a test set in a 7:3 ratio. The accuracy of the test set was 70.96%, indicating that the model was effective (Table 3).

**Table 3:** Recognition accuracy of the model.

Name	*n*	%
Model accuracy	2,023	70.96
Model error	828	29.04
Total	2,851	100

## 4 Discussion

This research endeavored to investigate the variances in the occurrence rates and associated risks of gross motor developmental lag in children below 3 years old. Concurrently, a decision-tree model was established to elucidate the importance of each factor causing gross motor developmental delay. The findings from this study furnished a solid foundation for an in-depth comprehension of the prevalence of motor developmental delays. Our findings showed that approximately 1.43% of children under the age of 3 years exhibited a low prevalence of gross motor developmental delay, consistent with our initial prediction. Moreover, the logistic regression analysis demonstrated that age, whether supplementary food was given, average time spent interacting with children, family type, feeding methods, mode of delivery, and birth order were the most significant predictors of gross motor developmental delay. The accuracy of the test set was 70.96%, indicating that the model was effective.

Utilizing the decision tree approach, our analysis revealed that age was the most significant factor affecting gross motor developmental delay among children under the age of 3 years. The likelihood of gross motor developmental delay increases with age. This finding has been confirmed in a number of studies (34, 35). Therefore, we should strengthen the monitoring of children’s GMS, with regular developmental assessments to identify potential gross motor delays. We found that there was no statistically significant difference between the sexes. However, there is no consensus on this conclusion (34, 36–38). Moreover, a child’s birth weight and gestation period are not associated with GMS. However, there is evidence that shorter gestational age is associated with gross motor developmental delays, and birth weight is significantly associated with motor development (39, 40). However, this study has a larger range of samples, and the results are more credible. To address this discrepancy, more research is needed in the future to explore it in depth.

This study established a positive link between exclusive breastfeeding and gross motor skills, with a multitude of studies affirming the clear-cut advantages of exclusive breastfeeding for child development (41–43). Exclusive breastfeeding duration as a factor was defined differently in all studies, and conflicting evidence was found regarding the role of exclusive breastfeeding (42, 43). Furthermore, we found that there was an increased prevalence of gross motor developmental delays in children with instrumental delivery compared to natural delivery. There are even studies showing that delivery circumstances had an association with attention-deficit hyperactivity disorder (44) and autism spectrum disorder (45). However, existing research findings are still uncertain regarding the precise impact of cesarean delivery on gross motor skills. For instance, certain studies have detected no substantial disparity in gross motor performance up to 12 months between children conceived through assisted reproductive technology and those in the control group (46–48).

This study did not find a correlation between maternal depression and children’s gross motor development. Overall, no studies have confirmed this association (49, 50). Furthermore, our research illuminated a positive correlation between GMS and average time spent interacting with children and no correlation between GMS and average parent–child reading time per day. A Canadian investigation determined that increased reading duration across various time points was considerably correlated with enhanced ASQ-3 scores in fine motor, gross motor, personal-social, and overall developmental domains over time (51). Therefore, more research is needed to explore this area in the future.

This research encountered several limitations. First, the cross-sectional methodology employed in this study restricted the ability to definitively infer causality. Second, the ASQ-3 was not designed for diagnosing gross motor developmental delays. Third, the data were obtained from the parents of the infants, who might lack comprehensive knowledge of their child’s precise condition, potentially leading to discrepancies between the reported data and the actual circumstances.

This research was endowed with multiple advantages. Initially, it boasted a substantial and representative participant pool. Moreover, it delved into the correlation between maternal gestational conditions and developmental delays.

## 5 Conclusion

Through the decision tree model, a total of seven critical influencing factors for gross motor developmental delay were screened. The top three factors in order of their importance were age, supplementary food, playtime, and family type, followed by feeding methods, mode of delivery, and birth order.

## Data Availability

The datasets presented in this article are not readily available because data is strictly confidential and cannot be shared. Requests to access the datasets should be directed to Yuxiang Xiong, yuxiangxiong2022@163.com.
